# The Antecedents and Consequences of Travelers’ Well-Being Perceptions: Focusing on Chinese Tourist Shopping at a Duty Free

**DOI:** 10.3390/ijerph16245081

**Published:** 2019-12-12

**Authors:** Hyunjoon Kim, Jinkyung Jenny Kim, Muhammad Asif

**Affiliations:** 1 The Department of Tourism Management, The College of Business Administration, Dong-A University, Busan 49236, Korea; hyunjoon@dau.ac.kr; 2 School of Hotel and Tourism Management, Youngsan University, Busan 48015, Korea; 3 School of Public Affairs, Zijingang Campus, Zhejiang University, Hangzhou 310058, China; asif.ma015@gmail.com

**Keywords:** travelers’ experiences, duty free shop, well-being perceptions, brand attitude, brand preference, word-of-mouth

## Abstract

The purpose of this study was to explore the antecedents and consequences of travelers’ well-being perceptions in the context of a duty-free shop. For this, data were collected from 742 Chinese tourists who purchased goods at duty free shops while traveling in Korea within the past year using an online survey company’s system in China. The results indicated that pragmatic, hedonic, and sociability experiences have a positive influence on travelers’ well-being perceptions. In addition, travelers’ well-being perceptions aided the enhancement of brand attitude and brand preference, which in turn positively affected word-of-mouth. Novelty is the originality of this study as very few studies on this topic are presented in the extant literature and practical implications are also discussed.

## 1. Introduction

Rising incomes and interest in psychological well-being has driven a growing demand of Chinese outbound tourism [1]. China became the biggest feeder market in outbound tourism of the globe, with 150 million outbound visits in 2018, which is a 14.7% increase year on year [2]. Chinese people travel to various destinations all over the world, and the Republic of Korea (hereafter referred to as Korea) is one of their preferred travel destinations. Chinese tourists have consisted of 40.47% of the total foreign visitors to Korea in the past five years [3] and it is a remarkable number despite the restrictions imposed on Chinese group tours to Korea due to the conflict caused by the deployment of a US missile shield in 2017. Since China began to lift its travel ban on group tours to Korea, a dramatic increase of Chinese travelers in Korea is expected [4].

Along with the Korean Wave involving cultural items, such as TV dramas, K-pop, and food, shopping is a renowned determining motivation for foreigners to visit Korea [5,6]. In general, shopping is regarded as one of the universal activities that brings significant economic, psychological, and social benefits to tourists [7,8]. According to a survey conducted by the Korea Tourism Organization, 33.4% of Chinese visitors said shopping is their main reason for traveling to Korea and the various shopping items ranged from cosmetics to luxury goods [5,9]. As such, Chinese travelers contributed to huge retail spending, which resulted in Korea being the largest duty free shopping market around the world [10].

Duty free shops are retail outlet stores that are exempt from paying taxes. The Swiss research agency M1nd-set [11] reported that the Chinese are the leading nationality for duty free sales, with 13.2% in the world. Chinese consumers specifically look for duty free shops when traveling because they are perceived to provide high quality products and luxury brand items for cheaper prices than their home country [9]. Furthermore, it is noted that the products sold are perceived to be genuine since China strictly controls its goods. It is widely known that Korea is one of the most popular destinations for Chinese tourists who look for duty free shops. According to the Korea Duty Free Shops Association, Korean duty free shops had the highest revenues of $9.9 billion from January to June 2019 due to the large spending by Chinese travelers [12].

Travel is considered as a physical and psychological healthy pursuit, and many studies have demonstrated the significant contribution of travel experience to individuals’ overall wellness [13,14]. In other words, travel is a typical hedonic product and consuming hedonic products improves levels of satisfaction within the relevant life domains and consequently enhances perceptions of well-being [15]. Similarly, consumption experience has also been widely studied as a salient trigger of individuals’ overall sense of well-being [16,17]. Moreover, various benefits of luxury product consumption were found to be significant influencing factors of consumers’ subjective well-being perceptions [18]. Hence, in view of the main motive for Chinese travelling to Korea, understanding the impact of travelers’ experiences at a duty free shop on well-being perceptions would be necessary to establish a strategic plan. In addition, it would be worthwhile to determine the influence of formed well-being perceptions on brand attitudes and brand preferences, and consequently on word-of-mouth to enhance effective marketing tools to attract more Chinese travelers who love traveling and shopping.

In consideration of the large portion of Chinese among foreign arrivals in Korea, numerous studies were conducted on understanding the antecedents and consequences of Chinese travelers toward Korea as a travel destination [6,19]. For example, Chiu et al. [20] examined the cognitive and affective destination image in the formation of Chinese travelers’ satisfaction and loyalty toward Korea. Furthermore, research has studied Chinese traveling to Korea in the different scope of market segmentations that include medical tourism [21], casino [22], and K-culture [23]. These extant studies dealt with tourists’ perceptions from a cross-cultural perspective, motivations, and travel behaviors in a particular domain, such as casino, K-pop, or medical tourism, in order to propose effective ways to attract more Chinese travelers to Korea. However, Chinese travelers’ experiences in the context of the duty free industry remain unclear.

Although Korea is a loved neighboring country of Chinese outbound travelers, it merely represents 3.2% of China’s total outbound travel [2]. This implies that there is still much potential for Korea to attract Chinese travelers. The tourism industry in Korea has accordingly recognized China’s contribution and further opportunities for a number of years. More importantly, China is the key source market in driving the revenue of duty free shops in Korea with their lavish expenditure. Therefore, it would be meaningful to explore travelers’ experiences as triggers of well-being perceptions and the consequences for the flow from China to Korea in the duty free shopping context. With this respect, this present study aimed to investigate (1) the effect of travelers’ experiences on travelers’ well-being perceptions; (2) the influence of travelers’ well-being perceptions on the formation of brand attitudes, brand preferences, and word-of-mouth; (3) the impact of brand attitudes on brand preferences and word-of-mouth; and (4) the relationship between brand preferences and word-of-mouth. This is one of the first attempts focused on Chinese travelers’ experiences at a duty free shop in Korea in increasing well-being perceptions and we expect that the results of this study would contribute in academia through tapping this undiscovered area and create opportunities for Korean destination marketers, and duty free shop practitioners to establish customized strategies towards the Chinese target customer segment.

## 2. Literature Review

### 2.1. Travelers’ Experiences at a Duty Free Shop

Customer experience is described as personal response occurring in the mind of an individual while he or she is engaged on an emotional, physical, intellectual, or spiritual level [24] and it is often conceptualized as a mixture of cognitive and affective components [25]. These two central components in explaining customer experience were also portrayed as utilitarian and hedonic [26], and extrinsic and intrinsic [27]. The former dimension involves the viewpoint of a person’s information processing and the latter dimension encompasses emotions resulting from various interactions within certain environments. In this regard, Bagdare and Jain [28] defined customer experience in a retail shop as “the sum total of cognitive, emotional, sensorial, and behavioral responses produced during the entire buying process, involving an integrated series of interaction with people, objects, processes and environment in retailing”. As such, customer experience in retail stores was articulated as every element that encourages or inhibits individuals while she or he is interacting with a retailer, and numerous studies have provided evidence of the impact of customer experience that includes influences on customer satisfaction, behavioral intentions, sales, and the image of retail stores [29,30].

Meanwhile, customer experience in consumption behavior is generally measured with customer experience quality [31], customer experience index [32], utilitarian and hedonic shopping value [27], retail service quality [33], and smart shopping [34] in the context of the retail industry. For instance, Grewal, Levy, and Kumar [29] presented the theory of customer experience and empirically uncovered the important role of price, promotion, merchandise, supply chain, and location in influencing customer experience and behaviors in the retail environment. Lemke et al. [35] stressed the importance of the quality of the customer experience through identifying the effect of product quality, communication, and social images on relationship outcomes in the business-to-business (B2B) and business-to-consumer (B2C) contexts. Klaus and Maklan [31] proposed a customer experience quality (EXQ) scale, which is a multiple-item scale consisting of product experience, outcome focus, moments of truth, and peace of mind, and addressed its impact on customer satisfaction and loyalty.

In addition, customer experience was often studied based on the four underlying dimensions, which are pragmatic, hedonic, usability, and sociability experiences [36,37,38]. For example, Salehi et al. [39] developed the framework to identify the strong drivers of service quality from a customer experience perspective and verified that pragmatic, hedonic, usability, and sociability experiences are factors influencing service quality. Lorenzo-Romero, Constantinides, and Brünink [36] encompassed these four dimensions in the context of online communities and confirmed their roles in enhancing customer participation in co-creating activities. Zafir [40] asserted the significant role of customer experience on the success of business and tested the effect of pragmatic, hedonic, usability, and sociability experiences on brand loyalty. Nambisan [41] explored social media user experience and found that the prominent impact of the four underlying dimensions (i.e., pragmatic, hedonic, sociability, and usability experiences) on mental well-being. Even though these four sub-dimensions of customer experience were widely studied in diverse settings, including online retail, it has not been well captured in the offline retail context. Thus, we identified an opportunity to adopt these four underlying dimensions of customer experience (i.e., pragmatic experience, hedonic experience, sociability experience, the usability experience) in the field of the offline duty free industry.

First, pragmatic experience relates to the practical and utilitarian aspects of customer experience in acquiring and processing information about a product or service during interactions in a store [37]. That is, pragmatic experience reflects how customers found the experience in a shop helpful, useful, valuable, or worthwhile [38,42]. Hoffman and Novak [43] stated that pragmatic experience presents customers’ goal orientated mindset, and a duty free shop is a particularly known place for travelers with definite sets of goals in regards to a list of purchasing items [44]. Therefore, pragmatic experience at a duty free shop is essential and can be described as the extent to which customers obtain required knowledge and purchase their wish list of items successfully.

Second, hedonic experience involves an affective component of experience and encompasses emotions and feelings, such as boredom, excitement, fun, or frustration [38,45]. As such, hedonic experience is regarded as the intrinsic value of travelers’ experience. In the case of a retail environment, hedonic experience relates to the atmospheric and service cues that include pleasurable moments of truth and appeal to the multiple senses of sight, sound, smell, and touch [46]. Similarly, Jones et al. [47] and Park and Park [48] argued that retailers should create an immersive environment to energize individuals’ shopping experiences in order to foster relationships between customers and brands. Thus, hedonic experience at a duty free shop is considered an important travelers’ experience influencing their responses and behaviors.

Third, sociability experience captures how customers perceive welcoming, friendliness, inviting, and politeness aspects of service providers. Sociability is part of basic inborn personality attributes [49] and sociability experience involves a communication and customer–employee interaction in a store, forming emotional engagement and stimulating some reactions [50,51]. Therefore, customers’ higher levels of sociability experience are likely to build stronger ties with a particular supplier and induces positive behavior. Wardono et al. [49] asserted that customers today have a tendency to select shopping places where their pleasure, through experiential socialization, is met. Hence, sociability experience at a duty free shop is one of the critical experiences that travelers extract from an atmosphere and service providers through their interactions with employees and the environment.

Last, usability presents the extent to which individuals can successfully complete tasks with effectiveness and efficiency [52]. Thus, usability experience depends on the overall environment and whether it is easy or difficult, confusing or not confusing, consistent or inconsistent, stressful or not stressful, simple or complicated, and tiring or not tiring [41]. In this respect, usability in a computer-mediating space is often measured by the ease of use and clarity of the technological features [37]. A duty free shop comprises several functional motivations of travelers that include convenience, product assortment, and quality shopping [53]. Hence, usability experience in a duty free shop reflects an easy flow of customer traffic, simple layout, visual appeal, and an array of products or categories that allow customers to be stress free and not bored or confused.

### 2.2. Travelers’ Well-Being Perceptions

A duty free shop is an experiential service setting where travelers shop for a wide range of products, interacting with employees at different stores, and purchase various products or services in the environment of modern and luxurious interiors, famous brands, and fashionable trends [48,54]. Duty free shops are no longer limited to the airport where guests may experience time pressure, and are often found in the city center in Korea [12]. Thus, Chinese travelers are more easily engaged with shopping motives around the country during their time in Korea.

The concept of well-being perceptions is increasingly drawing attention in many industries since customers today appreciate products and services that enhance their quality of life [55]. Grzeskowiak and Sirgy [56] illustrated well-being perceptions as “consumers’ perception of the extent to which a brand positively contributes to a quality of life enhancement”. Moreover, they argued that a positive perception of well-being involves overall happiness and higher satisfaction of his or her life. According to the bottom-up spillover theory, well-being perception is affected by satisfaction with all life domains and the theory was often adopted in order to explain such relationships as travel and consumption experiences affect concrete psychological domains [14,17,57]. Hence, the holistic consumption experience at a duty-free shop involves the combination of cognition, affect, and sense that would affect travelers’ subsequent responses. That is, shopping activities at a duty free shop would create an opportunity to enrich travelers’ well-being perceptions. Furthermore, purchasing luxurious products is known to elevate individuals’ perceptions of well-being and it has been verified across various settings in the hospitality and tourism industry [58,59]. In the meantime, a number of researches have confirmed the positive outcomes of well-being perceptions. For instance, the prominent role of well-being perceptions on brand love or loyalty [57,60] and customers’ behavior [61,62] were empirically found.

### 2.3. Brand Attitude and Brand Preference

Brand attitude was conceptualized as “a relative enduring, unidimensional summary evaluation of the brand that presumably energizes behavior” [63]. According to Thompson et al. [64], brand attitude encompasses cognitive and emotional components. In other words, it reflects the degree of customers’ information processing and how product or service providers create emotional ties with customers. On the other hand, Keller et al. [65] asserted that brand attitude is a function of brand attributes that result from brand benefits. That is, brand attitude is customers’ overall evaluation toward the brand and numerous scholars have presented the determinants, including aesthetic attributes, such as design [66], and utilitarian attributes, such as easy management [67]. In this regard, brand attitude has been extensively studied as an indispensable variable in a highly competitive market [68,69]. Adopting these notions into the context of the duty free industry, brand attitude toward a duty free shop would be formed through person-based variables and the shopping environments, including layout and interior design, that help travelers’ comfortable and enjoyable experience.

Together with brand attitude, brand preference was determined as one of the crucial variables in brand strategy. Hellier et al. [70] described brand preference as “the extent to which the customer favors the designated service provided by a certain company, in comparison to the designated service provided by other companies in his or her consideration set”. Brand preference is treated as a prominent component of brand loyalty [71] and is the conclusion of evaluations through a buying process and consumers’ attitude toward the brand [72,73]. As such, a brand preference can be formed through a bundle of products or services, and market attributes that lead customers to favor one brand over another. Yıldız and Akyol [73] explained that a brand preference is formulated due to various factors from customers’ perception, requirements, and to environmental, personal, social, and physiological reasons. Thus, understanding how a brand preference is built and the important role of brand preference, inducing customer behavior in the face of fierce competition of the duty free industry, should be recognized.

### 2.4. Word-of-Mouth

Westbrook [74] defined word-of-mouth as “informal communication directed at other consumers about the ownership, usage or characteristics of particular goods and services and/or their sellers”. The powerful marketing impact of word-of-mouth on consumer behavior and decision-making is well known as individuals greatly rely on opinions from people around them [75,76]. It is also recognized as a more effective marketing channel than commercial advertisements from a company standpoint and is considered fair and unbiased [77]. In this respect, word-of-mouth was examined as one of the essential customer behaviors in various contexts and many studies were conducted to identify the triggers of word-of-mouth, such as customers’ perceptions and brand-related variables [32,73,78].

### 2.5. Relationships among Study Variables

Travelers’ experiences have been extensively studied and their impact on travelers’ perceptions of well-being has been supported through much research. For example, Milman [79] explained that travelers’ experiences are not only related to physical activities, such as dining, flying, shopping, and riding, but also involve a wide range of cognitive activities. Accordingly, the author postulated the significant impact of travel experience on well-being perceptions and studied senior travelers’ psychological well-being by assessing the level of happiness pre and post tour. Sirgy et al. [17] surveyed 264 tourists and provided support for the strong association between travelers’ experiences and an overall sense of well-being. Kim et al. [61] measured cognition, emotion, and sensory experiences of airline travelers at airline lounges to examine the impact on well-being perceptions. Their results revealed that cognitive and sensory experiences are positively associated with the perceptions of airline travelers’ well-being.

Consumption experience has also been recognized as a vital predictor of individuals’ well-being. Schouten et al. [80] explained the concept of transcendent customer experiences (TCEs) as a flow generating long-lasting shifts in customers’ beliefs and attitudes and argued that enterprises should provide services that care about customers’ well-being. Hudders and Pandelaere [18] conducted a large-scale survey in Dutch-speaking Belgium and demonstrated that luxury product consumption experience increases customers’ subjective well-being. Roy et al. [81] studied customer experience in the smart technology-based retail context and confirmed that customer experience elevates customer-level outcomes, such as well-being. Park and Park [48] collected a survey from 305 customers of duty free shops at an airport and their analysis results indicated that if a duty free shop is convenient, its facilities are attractive, and the customer traffic line lines are functionally well arranged, shoppers will have greater effects on their emotional response. On parallel lines, the present study posited that the four underlying dimensions of customer experience would be significantly associated with travelers’ well-being perceptions:

**Hypothesis** **1 (H1).** *Travelers’ pragmatic experience has a positive influence on travelers’ well-being perceptions*.

**Hypothesis** **2 (H2).** *Travelers’ hedonic experience has a positive influence on travelers’ well-being perceptions.*

**Hypothesis** **3 (H3).** *Travelers’ sociability experience has a positive influence on travelers’ well-being perceptions.*

**Hypothesis** **4 (H4).** *Travelers’ usability experience has a positive influence on travelers’ well-being perceptions.*

Consumers generally purchase products or services that enhance their well-being needs [62] and customers’ perception of well-being is considered as a crucial trigger of brand attitude and brand preference. Accordingly, individuals’ well-being perceptions are generally proposed to increase customers’ brand love. For example, Ok et al. [60] explored the impact of well-being perceptions on customers’ enduring desire to maintain the relation with a particular brand in the domain of the coffee industry, and their results using 309 surveys demonstrated the positive effects in such a relationship. El Hedhli et al. [57] investigated the antecedents and consequences of customers’ well-being in the shopping mall context and confirmed the significant role of well-being in building loyalty toward a particular mall. In addition, well-being perceptions were verified to be the significant driver of customers’ behavioral intentions and behaviors, such as spreading positive word-of-mouth [61,62]. For instance, Kim et al. [14] conducted an empirical study based on a survey with 290 elderly tourists and provided evidence of the positive relationship between overall quality of life and behavioral intentions. Kim et al. [61] explained that travelers’ word-of-mouth communication was dependent on travelers’ well-being perceptions of an airline lounge. Similarly, Kim et al. [59] addressed the strong associations among airline travelers’ well-being perceptions, brand attachment, and word-of-mouth. On the basis of these theoretical foundations and empirical evidences, it is reasonable to expect the significant impact of travelers’ well-being perceptions on customer responses towards brand and behavior in the context of a duty free shop:

**Hypothesis** **5 (H5).** *Travelers’ well-being perceptions have a positive influence on brand attitude.*

**Hypothesis** **6 (H6).** *Travelers’ well-being perceptions have a positive influence on brand preference.*

**Hypothesis** **7 (H7).** *Travelers’ well-being perceptions have a positive influence on word-of-mouth.*

Percy and Rossiter [82] conceptualized brand attitude in that motive-anchored and stressed brand attitude involves an expected buying motive. Brand attitude indicates customers’ favorable or unfavorable responses that serve as an antecedent to customers’ behavior, such as willingness to purchase and loyalty [83,84]. As the correlation between attitude and affect was found in social psychology, brand attitude was verified as a predictor of brand preference. That is, a positive brand attitude results in a continuous preference of customers toward the specific brand [69]. For instance, Niedrich and Swain [85] provided evidence of the strong association between brand attitude and brand preference based on the data analysis collected from 231 participants. Chang and Liu [86] conducted the survey in three different service settings and analyzed 456 sets of data to test associations among brand attitude, equity, image, and preference. Their results showed brand attitude, which is the expression of customers’ evaluation of a brand contributed to increase brand attractiveness and association, and consequently build brand preference. In addition, brand attitude was verified in inducing customers’ word-of-mouth. Lau and Ng [68] explained that word-of-mouth is a form of customer response and demonstrated how individual and situational factors, and attitude toward a brand influencing word-of-mouth subject to samples in Singapore and Canada. Karjaluoto et al. [72] described the significant relationship between brand attitude and brand love, and confirmed its positive effect on favorable word-of-mouth. That is to say, it is generally believed that a favorable attitude results in brand preference and a positive word-of-mouth:

**Hypothesis** **8 (H8).** *Brand attitude has a positive influence on brand preference.*

**Hypothesis** **9 (H9).** *Brand attitude has a positive influence on word-of-mouth.*

Many studies across the different contexts provided support that brand preference exerts positive effects on customers’ word-of-mouth [32,73,87]. For example, Hwang [88] collected the survey from 293 fine dining customers in the United States and discovered that brand preference leads customers to spread positive word-of-mouth. Based on the result analysis, the author emphasized that brand preference is the key antecedent of patron behavior. Jalilvand et al. [89] examined the consequences of brand preference in the context of the restaurant industry based on a sample of 360 respondents and found the strong association between brand preference and customers’ word-of-mouth. In the study conducted by Yıldız and Akyol [73], the influence of brand preference on customers’ positive word-of-mouth was empirically verified by an analysis using 1000 surveys collected. They further asserted the importance of emotional ties that would encourage customers to prefer a certain brand even against the lower price or value added of the other similar quality brands. Jiang et al. [87] incorporated the theory of brand experience to examine the effect of customer experience on brand preference in the retailing context. Their results, based on statistical analysis using 710 sets of data, uncovered that customers with a high degree of certain brand preference are likely to recommend the brand to others through emotional responses.

**Hypothesis** **10 (H10).** *Brand preference has a positive influence on word-of-mouth.*

### 2.6. Proposed Model

**Figure 1:**
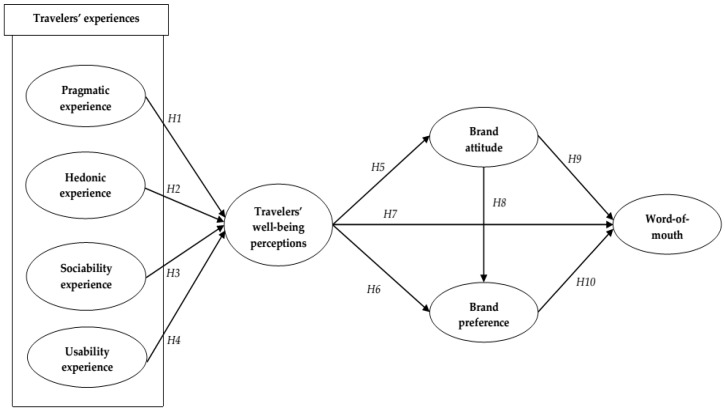
Proposed conceptual model. Note: H = hypothesis.

The conceptual model is proposed to grasp the relative influence of travelers’ experiences in increasing well-being perceptions and subsequent outcome variables, which involve eight constructs (see Figure 1). Ten hypotheses were constructed in order to examine the interrelationships among study variables that would enhance our understanding of antecedents and consequences of Chinese travelers’ well-being perceptions in the context of a duty free shop.

## 3. Methodology

### 3.1. Data Collection

The survey questionnaire was distributed to Chinese using an online survey company’s system in China, which is considered one of the biggest online panel survey companies in mainland China. There are more than about 2.5 million panelists in the online survey company’s system. In addition, there are three screening questions to guarantee the validity of samples. First, only respondents 18 years of age or older participated in this survey. Second, they had to have visited Korea at least once or more within the past year. Third, they had to have purchased goods at duty free shops during their trip. A total of 1000 samples were collected and 258 of them were removed due to visual inspection and the Mahalanobis distance check. Finally, 742 usable responses remained for further analysis, with a response rate of 74.2% (please see Table 1 and Table 2 for more details). Moreover, we applied Harman’s one-factor test to overcome the possibility of method bias. The total variance explained by a single factor was 19.68%, which is less than 50%, showing no bias issue [90].

### 3.2. Measures

Multi-item scales that had already been validated and widely adopted were revised to fit the shopping industry. Eight constructs were employed in the current study. Travelers’ experiences consisted of four sub-dimensions, including pragmatic, hedonic, sociability, and usability experiences, and were measured with 16 items adapted from Nambisan and Watt [37]. Travelers’ well-being perceptions were measured with three items used by Grzeskowiak and Sirgy [56] and Hwang and Lee [91]. Brand attitude was measured with six items from Hwang and Lyu [92] and Mitchell and Olson [93]. Brand preference was measured with three items from Hellier et al. [70] and Kim et al. [94]. Lastly, word-of-mouth was measured using three items from Hennig-Thurau et al. [95] and Hwang and Choi [96]. The questionnaire used a five-point Likert-type scale (See Appendix A for more details), anchored from strongly disagree (1) to strongly agree (5).

### 3.3. Demographic Characteristics

Table 1 provides the detailed profile of survey respondents. Among the respondents (*n* = 742), 44.5% (*n* = 330) were male while 55.5% (*n* = 412) were female. The survey respondents’ mean age was 32.76 years old. With regard to the level of education, the highest percentage category of respondents was a bachelor’s degree (*n* = 464, 62.5%). In addition, most respondents were married (*n* = 621, 83.7%). In the case of occupation, survey respondents reported that 63.9% (*n* = 474) were a company employee, self-employed (*n* = 53, 7.1%), sales/service (*n* = 15, 2.0%), student (*n* = 13, 1.8%), civil servant (*n* = 57, 7.7%), professional (*n* = 122, 16.4%), and other (*n* = 8, 1.0%). Lastly, for yearly household income, the majority of the respondents earned over US$27,771 to US$37,000 (*n* = 179, 24.1%).

Table 2 indicates the profile of the survey respondents’ travel characteristics. In terms of the number of visits to Korea, 38.1% (*n* = 283) had already visited Korea twice, followed by three times (*n* = 174, 23.5%). When respondents were asked about their main purpose of the travel, the largest category was leisure (*n* = 666, 89.8%). In addition, with regard to the length of stay, the highest percentage of respondents was three nights (*n* = 248, 33.4%), followed by four nights (*n* = 223, 30.1%). The largest group was accompanied by family or relatives (*n* = 397, 53.5%) when visiting Korea. Lastly, respondents spent an average US$2560 for shopping.

## 4. Data Analysis

### 4.1. Descriptive Statistics

The values of the mean, SD, and correlations of all the studied variables are illustrated in Table 3. The mean values ranged from 3.73 to 4.26, and the values of SD ranged from 86 to 97. It can be seen in Table 3 that the correlations among all studied variable are positive and significant. Table 3 further demonstrates the composite reliability (CR) values of the constructs and all of them were bigger than the 70 thresholds [97,98]. In addition, the average variance extracted (AVE) values for all of the constructs exceeded the recommended value of 0.50 [97,99], suggesting a high level of convergent validity. Lastly, the values of the square root of AVE for each construct were greater than the values of the inter-correlation between a pair of constructs, indicating discriminant validity [100].

### 4.2. Measurement Model

A confirmatory factor analysis (CFA) was used for assessing the measurement model [101]. According to the results of the CFA, the measurement model has an adequate fit to the data (χ^2^ = 717.922, NFI = 0.956, IFI = 0.979, CFI = 0.979, TLI = 0.974, RMSEA = 0.034). All these fit values fulfill the minimum criteria [102,103]. Table 4 shows the measurement items for each construct with their standardized loadings. As presented in Table 4, the standardized factor loading values for all the measurement items ranged from 0.733 to 0.885 and are greater than the cutoff value of 0.50, demonstrating convergent validity [104]. From the results of Table 4, we found that the composite reliability (CR) ranged from 0.83 to 0.90 for each factor. These values are greater than the recommended cutoff point of 0.60 [105] and confirmed the presence of inner consistency reliability among each construct [106].

### 4.3. Structural Equation Modeling (SEM)

**Figure 2:**
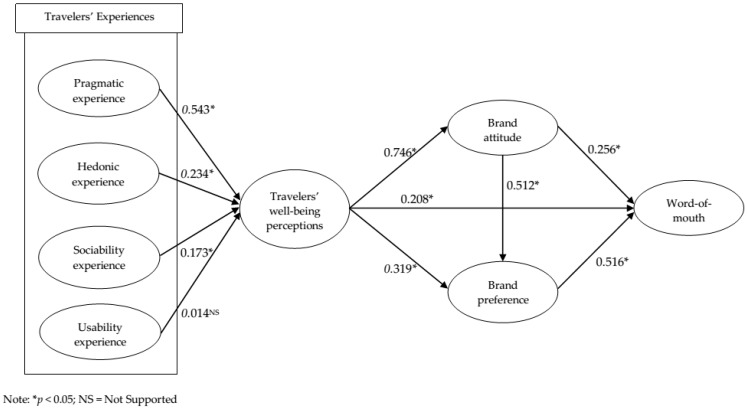
Standardized theoretical path coefficients.

SEM was utilized to evaluate the proposed conceptual model and the proposed theoretical hypotheses [107]. Table 5 and Figure 2 show the results of SEM with standardized regression weights. In addition, Table 5 displays the detailed hypotheses testing results. The overall fit of the proposed model was satisfactory (χ^2^ = 783.088, NFI = 0.953, IFI = 0.976; CFI = 0.976; TLI = 0.972, RMSEA = 0.036). The results of SEM analysis supported 9 of 10 hypotheses at a significance level of <0.05. Moreover, the t-values are also provided in Table 5 for better significance justification and these values should be greater than 1.96 [108] (see Table 5 and Figure 2).

## 5. Discussion and Implications

The present study focused on Chinese tourists in Korea in the context of the duty free industry and explored the antecedents and consequences of travelers’ well-being perceptions. Referring to the theoretical and empirical backgrounds, the conceptual model was proposed to assess the relationships among key study constructs. Then, an online survey was conducted with Chinese travelers who have experienced duty free shopping during their travel to Korea in the past year and a total of 742 responses, which captured the large number of participants, were used for data analysis. The results of the analysis identified the significant role of pragmatic, hedonic, and sociability experiences on travelers’ well-being perceptions. In addition, the statistical results revealed that travelers’ well-being perceptions enhanced brand attitude and brand preference, which positively affect word-of-mouth. This empirical study accordingly has the following meaningful theoretical and managerial implications.

First, this research is one of the first attempts exploring travelers’ experiences with a focus on Chinese travelers in Korea in the duty free shopping context. Chinese tourists toward Korea have been widely examined in various settings, such as medical tourism, casino, and K-culture [21,22,23]. However, duty free shopping has not been investigated enough, which is the main motive for Chinese is traveling to Korea. In this regard, this research is valuable as it provides information pertaining to how Chinese travelers’ well-being perceptions are built in the domain of the duty free industry and the consequences in inducing travelers’ behavior toward Korea as a travel destination. Moreover, this study incorporated sub-dimensions of the customer experience that have not been used in the offline retail context. Hence, this present study extended the literature of the customer experience by delivering evidence of the impact of such dimensions in the field of duty free shopping and provided a comprehensive understanding of Chinese travelers’ shopping experience in Korea.

Second, the analysis results discovered the prominent role of travelers’ experiences on well-being perceptions and is coherent with previous studies [17,48,81]. More specifically, our results showed the salient role of pragmatic experience (β = 0.543, *p* < 0.05) on travelers’ well-being perceptions and supported a study of Zafir [40], who confirmed the notion that pragmatic experience is a key determinant of overall quality experience. Jin et al. [1] explained that Chinese shopping practice is multi-faceted, including from simply looking for a souvenir and gift buying, to seeking authenticity, brand value, and pursuing high quality products. Therefore, operators of duty free shops should ensure their product range fits into their customers’ needs and offer the necessary information properly in order to capture more Chinese travelers and deliver a higher degree of their well-being perceptions. As such, listening to customers’ feedback would be the most effective and conducting an interview during the travelers’ waiting time at the gate before boarding or a minisurvey after their consumption experience at a duty free shop would be possible ways to collect travelers’ opinions and any desire for other product categories. Furthermore, Jin et al. [1] highlighted the importance of information and communication technologies (ICTs), such as shopping websites and mobile apps for Chinese travelers. Hence, practitioners should develop technology capacities to devise a customized shopping directory and applications with various functions in an online space. By doing so, Chinese people would obtain required knowledge and purchase their wish list of items successfully, and consequently improve their pragmatic experience, positively influencing well-being perceptions.

Third, hedonic experience (*β* = 0.234, *p* < 0.05) was found to be a predictor of travelers’ well-being perceptions, which is in accordance with the study results of Bagdare and Jain [28] and Verhoef et al. [30]. Jones et al. [47] argued that retailers create an immersive environment to energize individuals’ shopping experiences through stimulating customers’ auditory, olfactory, visual, and tactile faculties. Park and Park [48] demonstrated how the servicescape of an airport duty free shop influences customers’ emotional response. Therefore, the servicescape and sensory environment in the field of a duty free shop should be strengthened in order to improve travelers’ hedonic experience. Moreover, duty free shop practitioners continuously endeavor to be certain of Chinese travelers’ emotional engagement through atmosphere, social environment, and service interface in a duty free shop. In this respect, it is suggested to offer travelers a superior multi-sensory experience and differentiated places to stimulate intrinsic value. For example, creating a zone equipped with relaxing music, sofas, massage machines, or gaming facilities can be considered to enhance comfort, emotional security, and entertainment. Kim et al. [59] asserted that travelers’ experiences improve their quality of life, which is interchangeable in terms of well-being perception. They introduced enhanced programs and facilities at an airline lounge, such as a yoga session, and stressed the importance of wellness programs in enhancing travelers’ level of life quality. As such, a duty free shop can add touchpoints in relating wellness programs in these zones to increase the chances to have a higher level of hedonic experience and consequently well-being perceptions.

Fourth, the analysis results showed the positive impact of sociability experience (*β* = 0.173, *p* < 0.05) on travelers’ well-being perceptions. Employee attributes were often studied as a determining factor influencing customer experience in various service settings, and customer orientation and likeability were proven to be important sub-dimensions [109,110]. The level of customers’ sociability experience is determined by employees’ friendly, personal, polite, and inviting attributes and these can be improved through on-job training to increase their empathy, authentic service mindset, and attitude. A considerable number of researchers provided evidence that running a role play helps employees’ communication skills during the service encounter [111]. Thus, providing coaching sessions with diverse scenarios of service encounters is recommended to develop employees’ attributes to better deliver a sociability experience to travelers. In addition, despite the continuous efforts to improve language capabilities for Chinese shoppers across different regions, it is still noted as one of the biggest obstacles for many Chinese. For instance, Xu and McGehee [8] conducted in-depth phone interviews with Chinese tourists who traveled to the East Coast in the United States and their results demonstrated the importance of Chinese-speaking sales assistants to enhance Chinese’s shopping experience. Hence, more employees with Chinese-speaking competence would be helpful to provide a higher degree of customers’ sociability experience. Duty free practitioners accordingly should encourage employees’ ongoing language skill development and may consider deploying more fluent Chinese-speaking employees, especially during specific Chinese holidays. Enhancing Chinese shopping guides, directories, or signage in a store could be alternative solutions.

Fifth, contrary to our expectation, the relationship between the usability experience of Chinese travelers at a duty free shop and well-being perceptions was not statistically supported. We inferred that this is due to the different settings since many studies have supported the significant role of usability experience in the online space. For example, Salehi et al. [39] conducted an empirical study based on samples in Malaysia and illustrated that usability experience is one of the determinants of service quality in the online environment. A study conducted by Nambisan [41] investigated the association between social media user experience and mental well-being using 307 sets of data collected and the analysis results showed the positive impact of usability experiences on mental well-being. Since the customer experience in our study is mainly driven offline, it is worth noting that this study extended the current literature by confirming the different results in an online versus offline environment.

Sixth, the influence of travelers’ well-being perceptions on brand attitude (*β* = 0.746, *p* < 0.05), brand preference (*β* = 0.319, *p* < 0.05), and word-of-mouth (*β* = 0.208, *p* < 0.05) were statistically supported. These results supported the extant literature in the relationship between well-being perception and brand [57,60], and well-being perceptions and word-of-mouth [59,61]. In this regard, operators of duty free shops are encouraged to pay the utmost attention to travelers’ well-being perceptions and develop a strategic approach in inducing positive customer behaviors. Furthermore, the analysis result found that both brand attitude (*β* = 0.256, *p* < 0.05) and brand preference (*β* = 0.516, *p* < 0.05) were triggers of travelers’ word-of-mouth. Considering the fact that 52.3% of Chinese vacationers found information through friends, colleagues, and relatives [5], word-of-mouth is a powerful tool for a duty free shop in increasing the market share. Therefore, duty free shop practitioners should establish effective and innovative strategies to build travelers’ perceptions of well-being in order to drive Chinese travelers’ word-of-mouth. In a nutshell, the consequences of travelers’ well-being perceptions are crucial in the context of the duty free industry, and the perceptions of well-being should be treated as the key construct in promoting more favorable customer behavior.

## 6. Limitations and Future Research

Despite the aforementioned numerous implications, this study is not free from limitations. Numerous scholars addressed a number of factors impacting customer experience and behaviors. For instance, Jensen [112] asserted the different behaviors in the shopping context depending of the level of experience and Abdelmoteleb et al. [113] asserted the importance of understanding individuals’ psychology in explicating customer experience influencing their behavior. Choi and Park [44] confirmed the different customer behavioral intentions by gender in the context of a duty free shop. Therefore, it would be meaningful to examine the difference not only by demographic characteristics but also other factors, including customer culture, social class, or personality traits. That is, as the extension of the current study, it is recommended to test the moderating effect of these potential variables in the relationships among the proposed constructs.

## 7. Conclusions

Today, China is the largest source market for many countries in the tourism industry and its continuous leadership in travel abroad is anticipated as a basis of the promising outlook in a nation’s outbound tourism development [2]. In the same vein, China has been the number one feeder market of Korea for many years and shopping is the most compelling tool for the destination marketers of Korea to attract them [5,9]. Meanwhile, both travel and consumption experiences are extensively studied as they are related to individuals’ perceptions of well-being [15,16]. With this respect, the present study was developed to explore the antecedents and consequences of travelers’ well-being perceptions by understanding the roles of Chinese travelers’ experiences at a duty free. The conceptual model and 10 hypotheses were proposed based on a thorough literature review and all hypothesized associations among the study variables except the path linking usability experience and well-being perceptions were statistically supported. Overall, our study successfully captured the essence of the study objectives, with a focus on Chinese tourist shopping at a duty free in Korea, for the first time. The results of our study provided six principal findings that would also be insightful for a duty free shop in other regions that heavily rely on Chinese travelers. Despite some limitations, the findings of this study contribute to unearthing the structural relationships among key constructs in this under-researched field and provide practitioners with valuable information for developing effective strategies.

## Figures and Tables

**Table 1:** Profile of the survey respondents’ travel characteristics (*n* = 742).

Variable	*n*	Percentage
Gender		
Male	330	44.5
Female	412	55.5
Education Level		
High school diploma	170	22.9
Associate’s degree	35	4.7
Bachelor’s degree	464	62.6
Graduate degree	73	9.8
Marital Status		
Single	121	16.3
Married (including divorced and widow/widower)	621	83.7
Occupation		
Company employee	474	63.9
Self-employed	53	7.1
Sales/service	15	2.1
Student	13	1.8
Civil servant	57	7.7
Professional	122	16.4
Other	8	1.0
Yearly income		
Less than US$16,600	144	19.4
US$ 16,601–US$21,600	139	18.7
US$ 21,601–US$27,770	157	21.2
US$ 27,771–US$37,000	179	24.1
More than US$37,001	123	16.6
Mean Age = 32.76 years		

**Table 2:** Information about travel characteristics (*n* = 742).

Variable	*n*	Percentage
How many times have you visited Korea, including this tour?		
One time	165	22.2
Two times	283	38.1
Three times	174	23.5
Over four times	120	16.2
What is your main purpose of this tour?		
Leisure	666	89.8
Business	26	3.5
To meet friends or relatives	7	0.9
Shopping	43	5.8
How long did you stay in Korea?		
One night	7	0.9
Two nights	73	9.8
Three nights	248	33.4
Four nights	223	30.1
Over five nights	191	25.7
With whom did you travel?		
Alone	42	5.7
Friend	264	35.6
Association or Company	39	5.3
Family or Relatives	397	53.5
How much did you spend for shopping?	Average	US$2560

**Table 3:** Descriptive statistics and associated measures.

Construct	No. of Items	Mean (SD)	AVE	(1)	(2)	(3)	(4)	(5)	(6)	(7)	(8)
(1) Pragmatic experience	4	4.23 (0.89)	0.674	**0.821 ^a^**	0.768 ^b^	0.773	0.717	0.781	0.742	0.672	0.712
(2) Hedonic experience	4	4.10 (0.90)	0.672	0.590 ^c^	**0.820**	0.720	0.739	0.771	0.667	0.635	0.658
(3) Sociability experience	4	3.73 (0.97)	0.649	0.598	0.518	**0.806**	0.772	0.819	0.671	0.634	0.668
(4) Usability experience	4	4.05 (0.86)	0.655	0.514	0.546	0.596	**0.809**	0.778	0.679	0.652	0.683
(5) Well-being perceptions	3	4.24 (0.92)	0.701	0.610	0.594	0.671	0.605	**0.837**	0.690	0.655	0.718
(6) Brand attitude	6	4.26 (0.93)	0.698	0.551	0.445	0.450	0.461	0.476	**0.835**	0.753	0.776
(7) Brand preference	3	4.08 (0.89)	0.733	0.452	0.403	0.402	0.425	0.429	0.567	**0.856**	0.753
(8) Word-of-mouth	3	4.21 (0.88)	0.718	0.507	0.433	0.446	0.466	0.516	0.602	0.567	**0.847**
Goodness-of-fit statistics: χ^2^ = 717.922, *df* = 0.382; χ^2^/*df* = 1.879, *p* < 0.001, NFI = 0.956, IFI = 0.979, CFI = 0.979, TLI = 0.974, RMSEA = 0.034											

Note: SD = standard deviation; AVE = average variance extracted; NFI = normed fit index; IFI = incremental fit index; CFI = comparative fit index; TLI = Tucker–Lewis index; RMSEA = root mean square error of approximation. **^a^** Bold diagonal values are square root of AVE showing discriminant validity; **^b^** correlations are above the diagonal; **^c^** squared correlations are below the diagonal.

**Table 4:** Confirmatory factor analysis: Items and loadings.

Construct and Scale Items	Cronbach Alpha	Composite Reliability (CR)	Standardized Loadings ^a^
**Pragmatic Experience**	**0.849**	**0.892**	
Worthwhile			0.854
Useful			0.784
Valuable			0.843
Relevant			0.802
**Hedonic experience**	**0.843**	**0.891**	
Pleasing			0.793
Exciting			0.772
Deeply engrossing			0.864
Enjoyable			0.847
**Sociability experience**	**0.840**	**0.881**	
Friendly			0.733
Personal			0.779
Polite			0.781
Inviting			0.827
**Usability experience**	**0.869**	**0.883**	
Not tiring			0.805
Not stressful			0.820
Not confusing			0.811
Consistent			0.801
**Well-being perceptions**	**0.866**	**0.876**	
This duty free shop played an important role in my well-being.			0.842
This duty free shop met my overall well-being needs.			0.814
This duty free shop played an important role in enhancing my quality of life.			0.856
**Brand attitude**	**0.895**	**0.899**	
How would you characterize your attitude toward the duty free shop?			
I think my attitude toward the duty free shop is			
Unfavorable—Favorable			0.823
Dislike—Like			0.741
Bad—Good			0.790
Unpleasant—Pleasant			0.756
Poor quality—High quality			0.744
Unsatisfactory—Satisfactory			0.783
**Brand preference**	**0.841**	**0.892**	
When I want to shop, I consider this duty free shop a viable choice very often.			0.885
This duty free shop meets my shopping needs better than other comparable duty free shops.			0.871
I am interested in this duty free shop more than in other comparable duty free shops.			0.811
**Word-of-mouth**	**0.886**	**0.884**	
I said positive things about this duty free shop to others.			0.831
I recommended this duty free shop to others.			0.865
I encouraged others to visit this duty free shop.			0.845

Note: Bold values are showing constructs’ reliabilities. ^a^ standardized factor loadings.

**Table 5:** Standardized parameter estimates for the structural model.

Paths			Standardized Estimate	*t*-Value	Hypothesis
**H1:** Pragmatic experience	→	Well-being perceptions	0.543	4.498	Supported
**H2:** Hedonic experience	→	Well-being perceptions	0.234	2.589	Supported
**H3:** Sociability experience	→	Well-being perceptions	0.173	1.990	Supported
**H4:** Usability experience	→	Well-being perceptions	0.014	0.805	Not supported
**H5:** Well-being perceptions	→	Brand attitude	0.746	17.208	Supported
**H6:** Well-being perceptions	→	Brand preference	0.319	6.744	Supported
**H7:** Well-being perceptions	→	Word-of-mouth	0.208	5.032	Supported
**H8:** Brand attitude	→	Brand preference	0.512	9.917	Supported
**H9:** Brand attitude	→	Word-of-mouth	0.256	5.366	Supported
**H10:** Brand preference	→	Word-of-mouth	0.516	10.080	Supported
Goodness-of-fit statistics: χ^2^ = 783.088, *df* = 398, χ^2^/*df* = 1.968, *p* < 0.001, NFI = 0.953, IFI = 0.976, CFI = 0.976, TLI = 0.972, RMSEA = 0.036					

Note: H = hypothesis.

## Appendix A. Questionnaire

**Pre-screening questions**

**1. Did you visit Korea in previous one year? (1) No (2) Yes**

**2. When did you visit Korea? Year: __________ Month: ___________**

**3. Have you visited a duty free shop at a department store in Korea in your recent travel to Korea?**

  **Yes**  **Continue the survey**

  **No**   **End the survey**

**4. Please indicate your level of agreement with the following statement.**

	**Disagree**→**Neutral**→**Agree**				
**Pragmatic Experience**					
Worthwhile	1	2	3	4	5
Useful	1	2	3	4	5
Valuable	1	2	3	4	5
Relevant	1	2	3	4	5
**Hedonic experience**					
Pleasing	1	2	3	4	5
Exciting	1	2	3	4	5
Deeply engrossing	1	2	3	4	5
Enjoyable	1	2	3	4	5
**Sociability experience**					
Friendly	1	2	3	4	5
Personal	1	2	3	4	5
Polite	1	2	3	4	5
Inviting	1	2	3	4	5
**Usability experience**					
Not tiring	1	2	3	4	5
Not stressful	1	2	3	4	5
Not confusing	1	2	3	4	5
Consistent	1	2	3	4	5
**Well-being perceptions**					
This duty-free shop played an important role in my well-being.	1	2	3	4	5
This duty-free shop met my overall well-being needs.	1	2	3	4	5
This duty-free shop played an important role in enhancing my quality of life.	1	2	3	4	5
**Brand attitude** **How would you characterize your attitude toward the duty-free shop?** **I think my attitude toward the duty-free shop is…**					
Unfavorable—Favorable	1	2	3	4	5
Dislike—Like	1	2	3	4	5
Bad—Good	1	2	3	4	5
Unpleasant—Pleasant	1	2	3	4	5
Poor quality—High quality	1	2	3	4	5
Unsatisfactory—Satisfactory	1	2	3	4	5
**Brand preference**					
When I want to shop, I consider this duty-free shop a viable choice very often.	1	2	3	4	5
This duty-free shop meets my shopping needs better than other comparable duty-free shops	1	2	3	4	5
I am interested in this duty-free shop more than in other comparable duty-free shops	1	2	3	4	5
**Word-of-mouth**					
I said positive things about this duty-free shop to others.	1	2	3	4	5
I recommended this duty-free shop to others.	1	2	3	4	5
I encouraged others to visit this duty-free shop.	1	2	3	4	5

**5. How many times have you visited Korea including this tour?**

  (1) One time (2) Two times (3) Three times (4) Over four times

**6. What is your main purpose of this tour?**

  (1) Leisure (2) Business (3) To meet friends or relatives (4) Shopping (5) Others

**7. How long did you stay in Korea?**

  (1) One night (2) Two nights (3) Three nights (4) Four nights (5) Over five nights

**8. With whom did you travel?**

  (1) Alone (2) Friend (3) Association or Company (4) Family or Relatives (5) Others

**9. How much did you spend for shopping? ( )US$**
